# Supplementary material for: Discriminant Analysis of Pu-Erh Tea of Different Raw Materials Based on Phytochemicals Using Chemometrics
Source: Foods. 2022 Feb 25;11(5):680. doi: 10.3390/foods11050680 (PMC8909724; doi:10.3390/foods11050680)
Supplement: Supplementary file 1 [file foods-11-00680-s001.zip › foods-1601874-supplementary.pdf]

Supplementary Material

Table S1 The main phytochemicals of AT and TT[ab]. (Unit: mg/g)

Code[c]	WE	TPC	TFAAs	EGC	C	EGCG	EC	GCG	ECG	Caffeine	Theacrine
A1	503.87±3.59^{CDEFGHIJKLMNOPQRSTU}	248.27±1.58^{DEFGHIJKLMN}	26.08±0.26^{KLMNOPQRSTUVWX}	7.9±0.02^{YZabc}	3.47±0.36^{h}	50.4±0.51^{FGHIJKLMNOP}	20.25±0.24^{WX}	0.49±0.04^{bcd}	35.48±0.2^{EFGHIJKL}	42.54±0.31^{CDE}	2.67±0.07^{WX}
A2	486.73±7.63^{IJKLMNOPQRSTUV}	241.59±2.77^{FGHIJKLMNOPQRS}	30.15±0.36^{CDEFGHI}	11.48±0.02^{VWXYZ}	6.85±0.34^{PQRSTUVWXYZabcde}	40.19±0.23^{PQRSTUVWXY}	27.2±0.26^{MNOP}	1.28±0.04^{WXYZabcd}	31.49±0.2^{GHIJKL}	27.62±0.5^{OPQ}	2.79±0.04^{HI}
A3	504.07±4.25^{CDEFGHIJKLMNOPQRSTU}	233.52±1.2^{HIJKLMNOPQRSTUVWX}	24.14±0.04^{UVWXYZabcde}	11.16±0.09^{VWXYZa}	7.4±0.23^{MNOPQRSTUVWXYZabc}	32.88±0.25^{WXYZ}	30.43±0.26^{JK}	0.57±0.04^{abcd}	35.48±0.19^{EFGHIJKL}	27.38±0.41^{OPQ}	0.95±0.01^{VW}
A4	511.73±23.46^{BCDEFGHIJKLMNOPQ}	233.24±6.33^{HIJKLMNOPQRSTUVWX}	31.47±0.79^{ABCDE}	6.95±0.07^{Zabc}	3.77±0.43^{fgh}	90.29±0.37^{A}	25.32±0.06^{PQRST}	1.02±0.04^{YZabcd}	36.37±0.19^{EFGHIJKL}	32.69±0.27^{IJKL}	2.03±0.06^{NO}
A5	496.93±16.29^{EFGHIJKLMNOPQRSTUV}	229.96±0.45^{IJKLMNOPQRSTUVWXYZ}	32.73±0.35^{ABC}	7.43±0.04^{YZabc}	8.77±0.16^{JKLMNOPQRST}	42.83±0.39^{LMNOPQRSTUVWX}	30.4±0.26^{JK}	0.42±0.04^{d}	39.33±0.11^{CDEFGHIJKL}	27.71±0.44^{OPQ}	6.16±0.09^{L}
A6	513.67±7.58^{BCDEFGHIJKLMNOP}	230.51±5.36^{IJKLMNOPQRSTUVWXY}	31.06±0.63^{ABCDEFG}	13.18±0.36^{STUVW}	3.6±0.07^{gh}	61.3±0^{BCDEF}	61.75±0.26^{B}	3.38±0.04^{PQRS}	69.59±0.19^{A}	47.67±0.42^{A}	1.34±0.02^{I}
A7	485.90±1.30^{JKLMNOPQRSTUV}	219.56±1.35^{NOPQRSTUVWXYZa}	30.47±0.15^{BCDEFGH}	7.62±0.08^{YZabc}	3.92±0.34^{fgh}	44.83±0.2^{JKLMNOPQRSTUVW}	29.56±0.26^{JKL}	0.49±0.04^{bcd}	37.27±0^{EFGHIJKL}	30.5±0.2^{LMNO}	0.68±0.02^{G}
A8	477.75±9.69^{NOPQRSTUV}	229.01±4.31^{JKLMNOPQRSTUVWXYZ}	33.82±0.2^{A}	5.24±0.04^{c}	7.05±0.26^{NOPQRSTUVWXYZabcd}	31.39±0.05^{XYZ}	32.84±0.26^{I}	0.46±0.04^{cd}	32.65±0.19^{GHIJKL}	22.28±0.45^{STUVWXY}	2.43±0.17^{EF}
A9	527.25±7.39^{ABCDEFGHIJ}	225.05±3.26^{LMNOPQRSTUVWXYZa}	27.91±0.3^{GHIJKLMNOPQ}	6.72±0.04^{Zabc}	6.33±0.29^{TUVWXYZabcdef}	34.97±0^{STUVWXYZ}	30.87±0.26^{J}	0.47±0.04^{cd}	36.46±0.19^{EFGHIJKL}	25.33±0.29^{QRS}	0.64±0.01^{R}
A10	501.84±22.71^{DEFGHIJKLMNOPQRSTU}	222.69±1.2^{LMNOPQRSTUVWXYZa}	27.6±0.27^{HIJKLMNOPQR}	5.6±0.07^{bc}	7.85±0.2^{LMNOPQRSTUVWXYZa}	33.73±0.04^{VWXYZ}	42.4±0.26^{G}	0.64±0.04^{Zabcd}	35.81±0.19^{EFGHIJKL}	23.69±0.29^{RSTU}	1.71±0.01^{WX}

A11	$509.52\pm3.96^{BCDEFGHIJKLMNOPQRS}$	$243.06\pm0.45^{EFGHIJKLMNOPQ}$	$25.54\pm0.18^{MNOPQRSTUVWXYZ}$	20.08 ± 0.62^{KLMNO}	5.55 ± 0.21^{YZabcdefgh}	$45.84\pm0.19^{JKLMNOPQRSTU}$	25.07 ± 0.69^{QRST}	1.67 ± 0.04^{UVWXYZabcd}	$42.07\pm0.1^{BCDEFGHIJKL}$	23.71 ± 0.46^{RSTU}	0.63 ± 0^{S}
A12	$528.03\pm13.77^{ABCDEFGHI}$	$221.45\pm0.91^{MNOPQRSTUVWXYZa}$	32.09 ± 0.35^{ABCD}	17.95 ± 0.57^{LMNOPQRST}	12.69 ± 0.24^{F}	$44.93\pm0.39^{JKLMNOPQRSTUV}$	23.9 ± 0.03^{STU}	0.67 ± 0.04^{Zabcd}	32.86 ± 0.2^{FGHIJKL}	33.6 ± 0.28^{IJKL}	3.44 ± 0.03^{OP}
A13	$517.12\pm1^{BCDEFGHIJKLMN}$	273.75 ± 15^{ABCDE}	$25.72\pm0.13^{LMNOPQRSTUVWXY}$	16.64 ± 0.13^{NOPQRSTU}	$5.99\pm0.2^{VWXYZabcdefgh}$	$44.73\pm0.24^{JKLMNOPQRSTUVW}$	14.56 ± 1.1^{Z}	3.71 ± 0.36^{PQR}	29.05 ± 0.16^{IJKL}	46.22 ± 0.26^{AB}	0.13 ± 0.01^{U}
A14	$514.89\pm0.2^{BCDEFGHIJKLMNO}$	$236.24\pm7.5^{HIJKLMNOPQRSTUV}$	29.37 ± 0.56^{DEFGHIJ}	8.08 ± 0.1^{XYZabc}	4.52 ± 0.23^{defgh}	$49.86\pm0.39^{FGHIJKLMNOP}$	26.72 ± 0.26^{NOPQ}	0.66 ± 0.04^{Zabcd}	36.15 ± 0.19^{EFGHIJKL}	31.58 ± 0.3^{JKLMN}	0.22 ± 0.01^{W}
A15	$502.34\pm9.47^{CDEFGHIJKLMNOPQRSTU}$	$240.06\pm5.3^{GHIJKLMNOPQRST}$	33.77 ± 0.71^{A}	6.22 ± 0.02^{abc}	5.06 ± 0.12^{cdefgh}	27.75 ± 0.39^{Z}	25.56 ± 0.03^{PQRS}	1.4 ± 0.04^{VWXYZabcd}	30.75 ± 0.2^{HIJKL}	23.96 ± 0.64^{RST}	0.2 ± 0^{B}
A16	530.6 ± 0.7^{ABCDEFGH}	$215.26\pm5.86^{PQRSTUVWXYZa}$	28.81 ± 0.7^{EFGHIJKL}	13.78 ± 0.14^{QRSTUVW}	4.54 ± 0.16^{defgh}	55.34 ± 0.24^{CDEFGHIJ}	11.33 ± 1.11^{a}	1.15 ± 0.04^{XYZabcd}	24.02 ± 0.16^{LM}	45.04 ± 0.19^{ABC}	0.39 ± 0^{J}
A17	$522.99\pm9.86^{ABCDEFGHIJKL}$	$235.66\pm7.57^{HIJKLMNOPQRSTUVW}$	$28.25\pm0.33^{FGHIJKLMNO}$	19.96 ± 0.23^{KLMNO}	$6.79\pm0.14^{PQRSTUVWXYZabcde}$	$44.33\pm0.19^{JKLMNOPQRSTUVW}$	23.39 ± 0.7^{TU}	3.79 ± 0.36^{PQ}	39.13 ± 0.1^{CDEFGHIJKL}	24.01 ± 0.64^{RST}	0.32 ± 0^{C}
A18	543.42 ± 10.32^{ABC}	$238.44\pm1.28^{GHIJKLMNOPQRSTU}$	33.7 ± 0.42^{A}	18.25 ± 0.38^{LMNOPQRS}	$5.69\pm0.09^{WXYZabcdefgh}$	$48.25\pm0.19^{HIJKLMNOPQ}$	26.01 ± 0.69^{OPQR}	2.42 ± 0.04^{QRSTUVWXY}	37.69 ± 0.1^{CDEFGHIJKL}	23.56 ± 0.18^{RSTUV}	0.66 ± 0.02^{A}
A19	546.98 ± 4.44^{AB}	$245.77\pm3.17^{EFGHIJKLMNOP}$	31.42 ± 0.68^{ABCDEF}	17.28 ± 0.35^{MNOPQRSTU}	5.29 ± 0.09^{abcdefgh}	$53.07\pm0.19^{EFGHIJKLMNO}$	28.11 ± 0.35^{LMN}	1.97 ± 0.04^{STUVWXYZab}	$45.66\pm0.1^{ABCDEFGHIJKL}$	23.32 ± 0.48^{RSTUVWX}	0.56 ± 0.01^{H}
A20	542.86 ± 5.69^{ABCD}	$239.98\pm1.28^{GHIJKLMNOPQRST}$	$28.47\pm0.16^{EFGHIJKLMN}$	19.37 ± 0.53^{KLMNOP}	$5.57\pm0.19^{XYZabcdefgh}$	55.55 ± 0.19^{CDEFGHIJ}	28.91 ± 0.42^{KLM}	3.18 ± 0.04^{QRST}	54.22 ± 0.1^{ABCDEFGHIJ}	23.33 ± 0.57^{RSTUVWX}	0.59 ± 0.05^{S}
A21	538.15 ± 6.11^{ABCDE}	$245.14\pm2.51^{EFGHIJKLMNOP}$	$27.57\pm0.64^{HIJKLMNOPQRS}$	19.24 ± 0.51^{KLMNOP}	$5.9\pm0.19^{VWXYZabcdefgh}$	$53.66\pm0.19^{EFGHIJKLMN}$	28.65 ± 0.69^{KLMN}	8.5 ± 0.36^{JKL}	$46.97\pm0.1^{ABCDEFGHIJKL}$	23.38 ± 0.32^{RSTUVWX}	0.41 ± 0.05^{MN}
A22	534.49 ± 1.96^{ABCDEF}	$239.91\pm1.45^{GHIJKLMNOPQRST}$	29.85 ± 0.48^{CDEFGHI}	19.83 ± 0.09^{KLMNO}	$6.56\pm0.22^{RSTUVWXYZabcde}$	59.77 ± 0.19^{BCDEFGHI}	29.01 ± 0.69^{JKLM}	8.97 ± 0.36^{JK}	$53.67\pm0.1^{ABCDEFGHIJK}$	23.9 ± 0.38^{RST}	0.44 ± 0.07^{L}
A23	531.58 ± 0.69^{ABCDEFGH}	$227.64\pm3.49^{JKLMNOPQRSTUVWXYZa}$	$28.14\pm0.17^{GHIJKLMNOP}$	13.09 ± 0.2^{TUVWX}	$5.65\pm0.17^{WXYZabcdefgh}$	71.08 ± 0.17^{B}	51.5 ± 0.52^{D}	4.84 ± 0.36^{OP}	$51.48\pm0.1^{ABCDEFGHIJK}$	45.03 ± 0.54^{ABC}	0.59 ± 0.02^{NO}

A24	560.60 ± 6.33^{A}	$249.82\pm2.69^{DEFGHIJKLMN}$	30 ± 0.78^{CDEFGHI}	12.4 ± 0.31^{UVWXY}	$6.93\pm0.24^{OPQRSTUVWXYZabcd}$	$46.92\pm0.17^{JKLMNOPQRS}$	39.74 ± 0.52^{H}	6.37 ± 0.04^{MN}	$46.32\pm0.1^{ABCDEFGHIJKL}$	43.98 ± 0.08^{BCD}	2.05 ± 0.18^{K}
A25	$516.92\pm17.5^{BCDEFGHIJKLMN}$	197.64 ± 3.91^{a}	$27.02\pm0.43^{IJKLMNOPQRSTUV}$	13.08 ± 0.34^{TUVWX}	$5.93\pm0.13^{VWXYZabcdefgh}$	$50.08\pm0.17^{FGHIJKLMNOP}$	47.58 ± 0.52^{F}	7.23 ± 0.36^{LM}	40.41 ± 0.1^{CDEFGHIJKL}	37.69 ± 0.14^{FGH}	2.13 ± 0.13^{bc}
A26	$526.46\pm4.51^{ABCDEFGHIJK}$	206.8 ± 0.85^{VWXYZa}	$24.47\pm0.18^{RSTUVWXYZabcd}$	13.33 ± 0.12^{STUVW}	$5.78\pm0.1^{WXYZabcdefgh}$	$48.37\pm0.17^{HIJKLMNOPQ}$	49.44 ± 0.52^{EF}	6.24 ± 0.36^{MNO}	$41.55\pm0.1^{BCDEFGHIJKL}$	43.77 ± 0.41^{BCD}	3.7 ± 0.02^{m}
A27	532.63 ± 6.93^{ABCDEFG}	$230.39\pm3.69^{IJKLMNOPQRSTUVWXYZ}$	$25.12\pm0.26^{OPQRSTUVWXYZ}$	15.17 ± 0.64^{OPQRSTUV}	4.3 ± 0.18^{efgh}	97.73 ± 0.17^{A}	77.27 ± 0.52^{A}	12.67 ± 0.36^{GH}	28.76 ± 0.1^{JKL}	46.8 ± 0.67^{AB}	1.91 ± 0.04^{b}
A28	$530.33\pm18.13^{ABCDEFGH}$	$222.94\pm1.77^{LMNOPQRSTUVWXYZa}$	29.14 ± 0.48^{DEFGHIJK}	13.63 ± 0.35^{RSTUVW}	5.22 ± 0.24^{bcdefgh}	$38.84\pm0.17^{PQRSTUVWXYZ}$	38.57 ± 0.14^{H}	6.45 ± 0.36^{M}	36.72 ± 0.1^{EFGHIJKL}	45.48 ± 0.31^{ABC}	2.04 ± 0.3^{OP}
A29	$519.71\pm22.81^{ABCDEFGHIJKLM}$	208.3 ± 2.73^{UVWXYZa}	21.94 ± 0.29^{abcdefghi}	10.31 ± 0.02^{VWXYZabc}	$6.12\pm0.28^{UVWXYZabcdefg}$	$44.83\pm0.17^{JKLMNOPQRSTUVW}$	49.54 ± 0.52^{E}	4.88 ± 0.36^{NOP}	37.54 ± 0.1^{CDEFGHIJKL}	34 ± 0.44^{IJK}	1.07 ± 0.03^{ij}
A30	561.03 ± 12.49^{A}	$216.48\pm3.44^{OPQRSTUVWXYZa}$	$23.36\pm0.17^{WXYZabcdefg}$	13.62 ± 0.06^{RSTUVW}	$6.29\pm0.17^{TUVWXYZabcdef}$	$49.14\pm0.17^{GHIJKLMNOPQ}$	53.72 ± 0.52^{C}	6.44 ± 0.36^{M}	40.36 ± 0.1^{CDEFGHIJKL}	41.74 ± 0.36^{DE}	1.05 ± 0.13^{Z}
T1	468.59 ± 26.55^{STUVW}	203.59 ± 6.09^{XYZa}	$27.46\pm0.54^{HIJKLMNOPQRST}$	35.88 ± 1.76^{AB}	9.5 ± 0.52^{GHIJKLMNO}	$48.01\pm2.09^{IJKLMNOPQR}$	0.51 ± 0.01^{d}	3.05 ± 0.19^{QRSTU}	$51.6\pm6.27^{ABCDEFGHIJK}$	20.26 ± 1.34^{WXYZabc}	0.29 ± 0.02^{YZ}
T2	465.24 ± 4.54^{UVW}	$210.75\pm6.07^{STUVWXYZa}$	33.51 ± 1.29^{AB}	9.59 ± 0.89^{WXYZabc}	$6.6\pm0.34^{QRSTUVWXYZabcde}$	$41.67\pm5.59^{NOPQRSTUVWXY}$	0.89 ± 0.13^{cd}	32.41 ± 1.13^{A}	1.59 ± 0.11^{M}	35.87 ± 0.29^{GHI}	0.26 ± 0.02^{v}
T3	$474.38\pm1.58^{OPQRSTUVW}$	$236.73\pm3.79^{HIJKLMNOPQRSTUV}$	$23.28\pm0.66^{WXYZabcdefg}$	9.01 ± 0.62^{WXYZabc}	5.47 ± 0.14^{Zabcdefgh}	35.67 ± 8.08^{STUVWXYZ}	1.1 ± 0.16^{cd}	29.27 ± 0.52^{BC}	1.03 ± 0.07^{M}	27.87 ± 1.48^{OPQ}	0.42 ± 0.02^{g}
T4	$497.25\pm8.78^{EFGHIJKLMNOPQRSTUV}$	$244.08\pm2.03^{EFGHIJKLMNOP}$	21.07 ± 0.36^{efghijk}	18.22 ± 0.08^{LMNOPQRST}	$8.73\pm1.19^{JKLMNOPQRST}$	$44.94\pm3.47^{JKLMNOPQRSTUV}$	0.06 ± 0.02^{d}	2.35 ± 0.15^{QRSTUVWXY}	57.93 ± 12.49^{ABCDEFG}	17.48 ± 0.59^{bcdefg}	0.97 ± 0.04^{v}
T5	$486.77\pm1.23^{IJKLMNOPQRSTUV}$	281.01 ± 11.11^{ABC}	$22.75\pm1.22^{YZabcdefgh}$	24.08 ± 3.18^{GHIJK}	$7.26\pm1.06^{MNOPQRSTUVWXYZabc}$	40.3 ± 2.05^{PQRSTUVWXY}	0.19 ± 0.05^{d}	2.54 ± 0.2^{QRSTUVWX}	$47.96\pm8.23^{ABCDEFGHIJKL}$	16.77 ± 0.44^{defghi}	0.47 ± 0.11^{D}
T6	$504.34\pm10.31^{CDEFGHIJKLMNOPQRSTU}$	$258.55\pm6.19^{BCDEFGHIJ}$	20.83 ± 1.75^{fghijk}	29.9 ± 1.06^{CDEF}	10.06 ± 0.06^{GHIJKL}	$45.9\pm2.12^{JKLMNOPQRSTU}$	0.54 ± 0.06^{cd}	$1.91\pm0.09^{STUVWXYZabcd}$	61.22 ± 8.56^{ABCDE}	20.39 ± 0.66^{VWXYZab}	0.61 ± 0.08^{k}

T7	484.36±2.69^{LMNOPQRSTUV}	296.77±13.75^{A}	24.68±1.24^{RSTUVWXYZabc}	31.82±1.69^{ABCD}	9.55±0.41^{GHIJKLMN}	43.1±3.72^{KLMNOPQRSTUVWX}	0.53±0.08^{cd}	2.29±0.09^{RSTUVWXY}	51.94±12.44^{ABCDEFGHIJK}	20.86±0.6^{TUVWXYZa}	0.64±0.26^{Q}
T8	492.69±5.07^{GHIJKLMNOPQRSTUV}	236.4±5.29^{HIJKLMNOPQRSTUV}	21.03±1.42^{efghijk}	26.51±4.16^{EFGHI}	8.19±1.35^{KLMNOPQRSTUVW}	50.82±3.98^{FGHIJKLMNOP}	0.26±0.3^{d}	2.49±0.14^{QRSTUVWXY}	53.99±11.33^{ABCDEFGHIJK}	15.45±0.45^{fghi}	0.29±0.02^{l}
T9	507.28±2.99^{BCDEFGHIJKLMNOPQRST}	224.94±3.2^{LMNOPQRSTUVWXYZa}	21.42±1.34^{defghijk}	26.44±1.49^{FGHI}	9.02±0.54^{JKLMNOPQRS}	53.9±3.53^{DEFGHIJKLM}	0.55±0.28^{cd}	2.17±0.15^{STUVWXY}	54.88±9.64^{ABCDEFGHIJ}	14.07±0.48^{hij}	0.43±0.11^{o}
T10	466.73±10.34^{TUVW}	227.93±11.42^{JKLMNOPQRSTUVWXYZa}	18.51±0.18^{jkl}	9.13±0.49^{WXYZabc}	6.22±0.28^{STUVWXYZabcdefgh}	34.3±12.14^{UVWXYZ}	0.64±0.29^{cd}	30.68±0.41^{B}	1.19±0.85^{M}	32.18±3.19^{JKLMN}	0.32±0^{WX}
T11	515.08±6.19^{BCDEFGHIJKLMNO}	208.63±6.37^{UVWXYZa}	19.77±1.17^{hijkl}	20.27±3.24^{KLMN}	32.71±1.93^{AB}	65.75±3.75^{BCD}	18.86±1.16^{XY}	9.76±0.54^{IJ}	34.81±2.21^{EFGHIJKL}	31.32±2.08^{KLMN}	0.33±0.2^{p}
T12	510.74±2.63^{BCDEFGHIJKLMNOPQR}	236.53±6.9^{HIJKLMNOPQRSTUV}	20.92±0.56^{fghijk}	30.21±1.61^{CDEF}	11.72±0.34^{FGH}	50.35±4.43^{FGHIJKLMNOP}	0.5±0.34^{d}	2.2±0.15^{STUVWXY}	60.95±15.94^{ABCDE}	18.83±0.46^{Zabcde}	1.04±0.02^{g}
T13	511.47±0.54^{BCDEFGHIJKLMNOPQ}	256.7±19.51^{BCDEFGHIJK}	19.14±0.71^{ijkl}	30.62±0.06^{BCDEF}	11.22±0.36^{FGHIJ}	55.46±2.94^{CDEFGHIJ}	0.38±0.17^{d}	2.31±0.12^{QRSTUVWXY}	64.2±9.15^{ABC}	16.82±0.56^{defghi}	0.64±0.36^{s}
T14	497.62±7.11^{EFGHIJKLMNOPQRSTUV}	272.15±26.82^{ABCDEF}	24.31±1.48^{TUVWXYZabcd}	25.76±1.53^{FGHIJ}	8.11±0.3^{KLMNOPQRSTUVWXY}	42.16±3.62^{MNOPQRSTUVWXY}	0.22±0.03^{d}	2.34±0.16^{QRSTUVWXY}	49.71±12.79^{ABCDEFGHIJKL}	17.42±0.54^{bcdefg}	0.16±0.07^{K}
T15	490.85±0.36^{HIJKLMNOPQRSTUV}	224.86±10.94^{LMNOPQRSTUVWXYZa}	20.62±0.53^{ghijk}	28.74±1.41^{CDEFG}	8.72±0.36^{JKLMNOPQRST}	43.25±3.4^{KLMNOPQRSTUVWX}	0.08±0.07^{d}	2.43±0.11^{QRSTUVWXY}	48.18±11.11^{ABCDEFGHIJKL}	15.04±0.41^{fghi}	0.32±0.12^{a}
T16	503.34±7.57^{CDEFGHIJKLMNOPQRSTU}	222.34±5.55^{MNOPQRSTUVWXYZa}	17.28±0.79^{lm}	28.28±1.48^{CDEFG}	10.13±0.55^{FGHIJKL}	45.8±2.88^{JKLMNOPQRSTU}	0.56±0.17^{cd}	1.98±0.14^{STUVWXYZab}	55.73±10.53^{ABCDEFGHI}	16.65±0.63^{defghi}	0.39±0.09^{e}
T17	497.08±0.48^{EFGHIJKLMNOPQRSTUV}	225.87±6.51^{KLMNOPQRSTUVWXYZa}	22.62±0.62^{YZabcdefgh}	28.41±0.89^{CDEFG}	8.71±0.27^{JKLMNOPQRST}	42.84±1.63^{LMNOPQRSTUVWX}	0.54±0.05^{cd}	2.11±0.1^{STUVWXYZ}	51.07±5.44^{ABCDEFGHIJK}	16.17±0.51^{efghi}	0.34±0.04^{T}
T18	456.41±6.28^{VW}	214.88±4.64^{PQRSTUVWXYZa}	25.06±1.16^{PQRSTUVWXYZa}	30.58±0.64^{CDEF}	9.36±0.33^{GHIJKLMNOP}	40.75±0.98^{PQRSTUVWXY}	0.44±0.06^{d}	2.41±0.07^{QRSTUVWXY}	52.98±4.07^{ABCDEFGHIJK}	23.49±0.61^{RSTUVW}	0.3±0^{YZ}
T19	476.71±18.45^{NOPQRSTUV}	209.56±15.3^{TUVWXYZa}	29.31±0.53^{DEFGHIJ}	10.63±0.79^{VWXYZab}	4.52±0.89^{defgh}	34.69±4.47^{TUVWXYZ}	0.99±0.04^{cd}	29.12±1.34^{C}	0.93±0.05^{M}	37.56±0.95^{FGH}	0.26±0.02^{o}

T20	496.46±3.69^{FGHIJKLMNOPQRSTUV}	261.93±10.04^{BCDEFGH}	21.06±0.76^{efghijk}	27.29±0.12^{CDEFGHI}	9.57±0.58^{GHIJKLMN}	41.13±3.52^{OPQRSTUVWXY}	0.31±0.15^{d}	2.13±0.22^{STUVWXYZ}	56.97±11.89^{ABCDEFGH}	17.96±0.18^{abcdef}	0.09±0.01^{r}
T21	484.76±7.1^{LMNOPQRSTUV}	242.72±11.89^{EFGHIJKLMNOPQR}	14.95±0.91^{m}	30.6±0.2^{BCDEF}	11.92±0.48^{FG}	40.06±1.49^{PQRSTUVWXY}	0.23±0.11^{d}	2.12±0.15^{STUVWXYZ}	67.47±7.25^{AB}	26.2±0.9^{PQR}	0.07±0.01^{u}
T22	504.05±4.32^{CDEFGHIJKLMNOPQRSTU}	295.64±16.52^{A}	24.46±0.34^{RSTUVWXYZabcd}	29.32±1.93^{CDEF}	10.62±0.59^{FGHIJK}	42.63±3.11^{MNOPQRSTUVWX}	0.5±0.02^{d}	2.08±0.22^{STUVWXYZ}	52.35±10.93^{ABCDEFGHIJK}	18.03±0.72^{abcdef}	0.24±0.05^{P}
T23	514.26±14.08^{BCDEFGHIJKLMNOP}	210.1±3.24^{TUVWXYZa}	27.35±0.16^{HIJKLMNOPQRST}	36.51±2.57^{A}	11.67±0.77^{FGHI}	48.89±6.26^{GHIJKLMNOPQ}	1.31±1.23^{bcd}	2.48±0.15^{QRSTUVWXY}	64.13±23.99^{ABCD}	27.49±1.36^{OPQ}	0.11±0.07^{M}
T24	433.44±7.22^{W}	201.09±15.33^{YZa}	26.39±2.34^{JKLMNOPQRSTUVW}	6.56±0.39^{Zabc}	3.95±0.08^{fgh}	30.49±5.01^{YZ}	1.23±0.19^{cd}	23±1.55^{DE}	1.05±0.11^{M}	34.79±0.36^{HIJ}	0.59±0.11^{k}
T25	488.9±0.53^{IJKLMNOPQRSTUV}	239.88±2.7^{GHIJKLMNOPQRST}	27.03±0.96^{IJKLMNOPQRSTUV}	7.64±0.47^{YZabc}	5.5±0.24^{Zabcdefgh}	46.34±5.9^{JKLMNOPQRST}	3.22±0.37^{b}	23.3±0.41^{D}	1.22±0.16^{M}	45.26±0.06^{ABC}	0.81±0.05^{j}
T26	467.34±4.66^{TUVW}	212.86±12.93^{QRSTUVWXYZa}	20.98±0.59^{efghijk}	8.93±0.69^{WXYZabc}	5.66±0.21^{WXYZabcdefgh}	36.09±9.18^{RSTUVWXYZ}	2.46±0.59^{bc}	21.53±0.54^{E}	1.38±0.12^{M}	40.27±0.09^{EF}	0.48±0.11^{XY}
T27	466.60±0.42^{TUVW}	199.33±5.42^{Za}	25.2±1.34^{OPQRSTUVWXYZ}	27.49±2.04^{DEFGH}	34.74±1.91^{A}	62.91±3.94^{BCDE}	18.97±0.93^{XY}	13.74±0.9^{G}	33.25±2.22^{FGHIJKL}	33.96±1.89^{IJK}	0.42±0.01^{ef}
T28	470.00±14.51^{RSTUVW}	216.21±4.41^{OPQRSTUVWXYZa}	22.4±0.26^{Zabcdefgh}	28.07±1.23^{CDEFG}	34.63±1.48^{A}	60.06±2.65^{BCDEFGH}	17.78±0.78^{Y}	15.25±0.67^{F}	27.36±1.2^{KLM}	32.3±1.52^{JKLM}	0.1±0.01^{c}
T29	479.87±3.18^{MNOPQRSTUV}	230.77±3.96^{HIJKLMNOPQRSTUVWXY}	18.37±1.71^{kl}	17.51±0.97^{MNOPQRST}	27.94±1.51^{D}	65.67±3.76^{BCD}	18.09±1.06^{Y}	11.19±0.64^{HI}	31.43±1.8^{GHIJKL}	33.26±2^{IJKL}	0.2±0^{n}
T30	492.72±5.38^{GHIJKLMNOPQRSTUV}	239.01±19.8^{GHIJKLMNOPQRSTU}	27.2±0.9^{IJKLMNOPQRSTU}	15.11±0.4^{OPQRSTUV}	9.23±0.17^{HIJKLMNOP}	44.15±3.51^{JKLMNOPQRSTUVW}	0.09±0.01^{d}	2.44±0.19^{QRSTUVWXY}	51.63±11.77^{ABCDEFGHIJK}	14.61±0.57^{ghij}	0.42±0^{e}
T31	522.48±7.01^{ABCDEFGHIJKL}	252.45±4.44^{CDEFGHIJKLM}	23.45±0.34^{WXYZabcdefg}	20.02±1.26^{KLMNOP}	7.44±0.47^{MNOPQRSTUVWXYZabc}	44.55±4.43^{JKLMNOPQRSTUVW}	0.26±0.08^{d}	1.94±0.12^{STUVWXYZabc}	49.71±13.33^{ABCDEFGHIJKL}	14.69±0.43^{ghi}	0.98±0.05^{q}
T32	485.36±0.83^{KLMNOPQRSTUV}	204.59±7.42^{WXYZa}	21.69±1.48^{bcdefghij}	22.25±4.34^{IJKLM}	8.59±1.49^{KLMNOPQRSTU}	55.09±4.97^{DEFGHIJK}	0.24±0.16^{d}	1.79±0.17^{TUVWXYZabcd}	61.04±16.23^{ABCDE}	13.7±0.45^{ij}	0.05±0.03^{ef}

T33	486.73±12.16^{IJKLMNOPQRSTUV}	210.45±10.5^{STUVWXYZa}	23.89±0.67^{VWXYZabcdef}	21.04±1.22^{JKLMN}	15.4±0.37^{E}	67.23±1.55^{BC}	22.3±0.53^{UV}	8.62±0.5^{JKL}	40.26±1.25^{CDEFGHIJKL}	38.13±0.82^{FG}	0.35±0.01^{hi}
T34	483.50±2.73^{LMNOPQRSTUV}	267.92±12.27^{ABCDEFG}	24±0.75^{VWXYZabcdef}	15.38±0.91^{NOPQRSTUV}	8.44±0.36^{KLMNOPQRSTUV}	41.11±2.08^{OPQRSTUVWXY}	0.14±0.14^{d}	2±0.11^{STUVWXYZa}	47.43±7.11^{ABCDEFGHIJKL}	14.16±0.42^{hij}	0.17±0°
T35	528.13±7.93^{ABCDEFGHI}	260.76±10.52^{BCDEFGHI}	23.3±0.31^{WXYZabcdefg}	24.09±0.77^{GHIJK}	9.82±0.45^{GHIJKLM}	46.45±2.16^{JKLMNOPQRST}	0.26±0.05^{d}	1.93±0.21^{STUVWXYZabc}	47.16±6.28^{ABCDEFGHIJKL}	14.08±0.52^{hij}	0.49±0^{p}
T36	478.75±16.48^{MNOPQRSTUV}	211.72±20.41^{RSTUVWXYZa}	20.54±0.12^{ghijk}	32.11±2.27^{ABCD}	9.16±0.53^{HIJKLMNOPQ}	41.59±4.17^{OPQRSTUVWXY}	0.36±0.05^{d}	2.53±0.15^{QRSTUVWX}	50.61±13.88^{ABCDEFGHIJKL}	21.6±0.21^{TUVWXYZ}	0.23±0^{d}
T37	466.10±0.07^{TUVW}	197.42±4.48^{a}	21.73±1.99^{bcdefghi}	18.77±0.59^{KLMNOPQRS}	29.87±1.47^{CD}	61.23±1.24^{BCDEFGHI}	22.2±1.37^{UVW}	9.01±0.55^{JK}	37.44±2.22^{DEFGHIJKL}	28.99±1.7^{NOP}	0.23±0.01^{k}
T38	486.95±0.29^{IJKLMNOPQRSTUV}	268.46±15.21^{ABCDEFG}	20.92±0.57^{fghijk}	22.32±1.68^{IJKLM}	34.12±2.54^{AB}	59.75±4.99^{BCDEFGHI}	27.71±2.07^{LMNO}	7.69±1.18^{KLM}	41.96±3.29^{BCDEFGHIJKL}	29.35±2.39^{MNOP}	0.2±0.01^{h}
T39	478.72±3.58^{MNOPQRSTUV}	246.89±6.75^{EFGHIJKLMNO}	20.87±0.17^{fghijk}	22.67±1.43^{HIJKL}	31.67±1.71^{BC}	60.73±5.37^{BCDEFG}	24.71±1.56^{RST}	7.29±1.23^{LM}	36.32±2.36^{EFGHIJKL}	27.32±1.89^{OPQ}	0.4±0.35^{v}
T40	501.14±0.47^{EFGHIJKLMNOPQRSTU}	272.64±7.03^{ABCDEF}	23.16±0.87^{XYZabcdefg}	32.46±2.39^{ABCD}	10.57±0.77^{FGHIJK}	44.47±3.47^{JKLMNOPQRSTUVW}	0.37±0.05^{d}	2.58±0.3^{QRSTUVWX}	59.58±11.88^{ABCDEF}	23.1±1.19^{RSTUVWX}	0.62±0.03^{p}
T41	498.79±3.92^{EFGHIJKLMNOPQRSTU}	295.29±8.12^{A}	19.21±0.82^{ijkl}	18.82±1.13^{LMNOPQ}	27.53±1.58^{D}	56.11±1.53^{CDEFGHIJKL}	20.6±1.22^{VWX}	7.47±0.53^{LM}	38.34±2.27^{CDEFGHIJKL}	31.46±2^{KLMN}	0.65±0.12^{t}
T42	502.38±2.54^{CDEFGHIJKLMNOPQRSTU}	250.72±6.76^{CDEFGHIJKLMN}	23.93±0.6^{VWXYZabcdef}	32.78±2.35^{ABC}	9.55±0.68^{GHIJKLMN}	38.84±4.02^{PQRSTUVWXYZ}	0.34±0.02^{d}	2.69±0.3^{QRSTUVW}	47.14±13.48^{ABCDEFGHIJKL}	23.11±1.16^{RSTUVWX}	0.32±0^{e}
T43	483.64±1.58^{LMNOPQRSTUV}	240.23±5.11^{GHIJKLMNOPQRST}	23.23±0.96^{WXYZabcdefg}	24.21±1.61^{GHIJK}	7.96±0.37^{LMNOPQRSTUVWXYZ}	37.75±3.24^{QRSTUVWXYZ}	0.06±0.03^{d}	2.11±0.11^{STUVWXYZ}	51.59±14.66^{ABCDEFGHIJK}	20.24±0.45^{XYZabc}	0.2±0.01^{r}
T44	499.46±7.46^{EFGHIJKLMNOPQRSTU}	215.11±2.12^{PQRSTUVWXYZa}	26.04±2.43^{KLMNOPQRSTUVWX}	32.13±1.1^{ABCDE}	8.45±0.02^{JKLMNOPQRSTUVWX}	39.78±3.2^{PQRSTUVWXY}	0.16±0.09^{d}	2.89±0.22^{QRSTUV}	51.2±10.67^{ABCDEFGHIJK}	23.27±0.94^{RSTUVWX}	0.16±0.01^{h}
T45	503.75±18.35^{CDEFGHIJKLMNOPQRSTU}	279.28±13.27^{ABCD}	24.4±1.75^{STUVWXYZabcd}	26.7±4.34^{EFGHI}	7.76±1.13^{LMNOPQRSTUVWXYZab}	44.05±4.95^{JKLMNOPQRSTUVW}	0.28±0.02^{d}	2.78±0.28^{QRSTUV}	48.17±15.17^{ABCDEFGHIJKL}	17.12±0.58^{cdefgh}	0.66±0.09^{E}

T46	496.79±5.44^{EFGHIJKLMNOPQRSTUV}	215.37±10.75^{PQRSTUVWXYZa}	28.52±1.89^{EFGHIJKLM}	31.94±1.87^{ABCD}	9.13±0.27^{IJKLMNOPQR}	44.14±4.12^{JKLMNOPQRSTUVW}	0.48±0.09^{d}	2.74±0.09^{QRSTUVW}	55.07±13.63^{ABCDEFGHIJ}	21.75±0.48^{TUVWXYZ}	0.15±0.09^{UV}
T47	498.00±3.12^{EFGHIJKLMNOPQRSTU}	226.64±6.86^{KLMNOPQRSTUVWXYZa}	18.79±0.23^{ijkl}	26.97±0.44^{DEFGHI}	8.69±0.45^{JKLMNOPQRSTU}	35.47±2.45^{STUVWXYZ}	0.13±0.02^{d}	2.08±0.19^{STUVWXYZ}	52.21±10.73^{ABCDEFGHIJK}	20.56±0.76^{UVWXYZab}	0.11±0.1^{r}
T48	471.15±3.68^{QRSTUVW}	285.57±15.74^{AB}	21.56±0.51^{cdefghij}	30.19±1.21^{CDEF}	9.13±0.24^{IJKLMNOPQR}	40.19±1.97^{PQRSTUVWXY}	0.05±0.04^{d}	2.53±0.09^{QRSTUVWX}	53.89±7.85^{ABCDEFGHIJK}	19.47±0.36^{YZabcd}	0.58±0.02^{F}
T49	472.88±15.27^{PQRSTUVW}	253.79±10.5^{CDEFGHIJKL}	24.8±0.29^{QRSTUVWXYZab}	18.63±1.57^{LMNOPQR}	11.73±0.4^{FGH}	63.03±2.54^{BCDE}	17.7±0.72^{Y}	7.96±0.28^{KL}	30.36±1.32^{HIJKL}	33.81±1.79^{IJK}	0.39±0.06^{l}
T50	469.21±2.17^{STUVW}	206.96±2.25^{VWXYZa}	25.3±1.65^{NOPQRSTUVWXYZ}	14.11±1.4^{PQRSTUVW}	7.65±0.6^{LMNOPQRSTUVWXYZabc}	43.99±4.14^{JKLMNOPQRSTUVW}	0.2±0.01^{d}	2.31±0.11^{QRSTUVWXY}	39.61±9.88^{CDEFGHIJKL}	11.4±0.54^{j}	0.34±0.01^{fg}

(continue) Table S1 The main phytochemicals of AT and TT[ab]. (Unit: mg/g)

Code[c]	Asp	Ser	Glu	Theanine	Gly	Ala	Cys	Val	Ile	Leu	Tyr	Phe	Lys	His	Arg
A1	-	-	1.88±0.01^{EF}	9.89±0.01^{WX}	0.02±0^{IJKL}	0.21±0^{HIJ}	-	0.23±0^{KL}	0.05±0^{OPQRST}	0.08±0^{RSTU}	0.08±0^{Zab}	0.14±0^{jk}	0.09±0^{k}	0.11±0^{Y}	0.34±0^{c}
A2	-	-	2.08±0^{D}	13.34±0.01^{HI}	0.02±0^{GHIJ}	0.26±0^{D}	-	0.26±0^{I}	0.06±0^{JKLM}	0.1±0^{N}	0.1±0^{UVW}	0.29±0^{a}	0.1±0^{j}	0.07±0^{bc}	0.46±0^{R}
A3	-	-	1.83±0.02^{FG}	10.02±0.01^{VW}	0.02±0^{JKLM}	0.21±0^{IJK}	-	0.19±0^{VWXY}	0.02±0^{UV}	0.05±0^{bcde}	0.05±0^{g}	-	0.07±0^{opq}	0.09±0^{a}	0.47±0^{R}
A4	-	-	2.01±0.02^{DE}	11.81±0.08^{NO}	0.02±0^{JKLM}	0.23±0^{E}	-	0.22±0^{LMN}	0.05±0^{NOPQR}	0.09±0^{OPQ}	0.07±0^{cde}	0.19±0^{e}	0.09±0^{k}	0.11±0^{Y}	0.63±0^{L}
A5	-	-	1.88±0.02^{EF}	12.24±0.08^{L}	0.02±0^{HIJK}	0.22±0^{FGHI}	-	0.2±0^{PQR}	0.05±0^{OPQRST}	0.08±0^{RSTU}	0.07±0^{cde}	0.14±0^{ij}	0.08±0^{kl}	0.11±0^{Y}	0.65±0^{JK}
A6	-	-	2.02±0.02^{DE}	13.2±0.08^{I}	0.02±0^{LMNOP}	0.23±0^{EF}	-	0.21±0^{OP}	0.05±0^{OPQRST}	0.08±0^{STU}	0.07±0^{de}	0.15±0^{hi}	0.08±0^{kl}	0.07±0^{bc}	0.82±0.03^{E}

A7	-	-	2.01±0.02^{DE}	14.26±0.01^{G}	0.03±0^{GHI}	0.24±0^{E}	-	0.2±0^{QRST}	0.04±0^{QRSTU}	0.07±0^{VWX}	0.07±0^{ef}	0.14±0^{ij}	0.07±0^{nop}	0.08±0^{b}	0.66±0^{IJ}
A8	-	-	1.74±0.01^{FGHI}	14.94±0.01^{EF}	0.02±0^{LMNO}	0.22±0^{GHI}	-	0.19±0^{WXY}	0.02±0^{UV}	0.05±0^{bcde}	0.06±0^{fg}	0.1±0^{l}	0.06±0^{q}	0.06±0^{cde}	0.8±0^{EF}
A9	-	-	1.73±0.02^{FGHI}	10.85±0.08^{R}	0.02±0^{KLMN}	0.2±0^{JK}	-	0.22±0^{KLMN}	0.05±0^{OPQRS}	0.08±0^{QRST}	0.07±0^{ef}	0.16±0^{fg}	0.08±0^{kl}	0.11±0^{Y}	0.61±0^{M}
A10	-	-	1.51±0.02^{JKLMN}	9.91±0.08^{WX}	0.01±0^{NOPQRS}	0.16±0^{MNO}	-	0.19±0^{UVWXY}	0.03±0^{RSTU}	0.07±0^{WX}	0.07±0^{ef}	0.17±0^{f}	0.07±0^{nop}	0.06±0^{cde}	0.43±0^{STU}
A11	-	-	1.57±0.02^{IJKL}	10.57±0.08^{S}	0.02±0^{IJKL}	0.23±0^{EFG}	-	0.23±0^{KL}	0.05±0^{MNOPQ}	0.08±0^{RSTU}	0.08±0^{Zabc}	0.13±0^{k}	0.08±0^{lmn}	0.07±0^{bc}	0.52±0^{P}
A12	-	-	1.79±0.02^{FGH}	11.75±0.08^{OP}	-	0.18±0^{L}	-	0.21±0^{OP}	0.04±0^{PQRST}	0.07±0^{TUVW}	0.07±0^{ef}	0.11±0^{l}	0.08±0^{lmn}	0.08±0^{b}	0.09±0^{i}
A13	-	-	-	10.24±0.01^{U}	-	-	0.04±0^{KL}	0.18±0^{Y}	-	-	0.2±0^{FG}	0.73±0^{J}	0.7±0^{G}	0.41±0NO	0.31±0^{d}
A14	-	-	1.82±0.01^{FG}	10±0.08^{W}	0.02±0^{JKLM}	0.21±0^{HIJ}	-	0.28±0^{H}	0.08±0^{GHIJ}	0.1±0^{N}	0.09±0^{YZa}	0.24±0^{d}	0.12±0^{i}	0.11±0^{YZ}	0.44±0^{S}
A15	-	-	2.57±0.02^{C}	18.97±0.02^{B}	0.03±0^{DE}	0.28±0^{C}	-	0.21±0^{OP}	0.05±0^{LMNOP}	0.09±0^{OP}	0.24±0^{C}	0.75±0^{I}	0.44±0^{V}	0.46±0^{HI}	0.52±0^{P}
A16	-	-	2.16±0.02^{D}	12.91±0.01^{J}	0.03±0^{CDE}	0.24±0^{E}	0.04±0^{KLM}	0.21±0NO	0.08±0^{GHI}	0.09±0°	0.23±0^{D}	0.87±0^{C}	0.46±0^{U}	0.48±0^{F}	0.39±0^{YZ}
A17	-	-	2.88±0.02^{B}	17.63±0.01^{C}	0.04±0^{CDE}	0.32±0^{B}	0.06±0^{J}	0.33±0^{E}	0.14±0^{E}	0.15±0^{J}	0.33±0^{A}	1.18±0^{A}	0.62±0^{L}	0.74±0^{A}	0.68±0^{H}
A18	-	-	3.75±0.02^{A}	20.79±0.01^{A}	0.04±0^{C}	0.38±0^{A}	0.05±0^{K}	0.24±0^{J}	0.09±0^{FG}	0.13±0^{K}	0.32±0^{A}	0.85±0^{D}	0.5±0^{S}	0.49±0^{E}	0.69±0^{H}
A19	-	-	2.04±0.02^{DE}	13.39±0.08^{H}	0.04±0^{CDE}	0.25±0^{D}	-	0.07±0^{f}	0.03±0^{RSTU}	0.05±0^{bcde}	0.19±0^{HI}	-	0.41±0^{W}	0.47±0^{GH}	1.16±0^{C}

A20	-	-	-	10.68±0.01^{S}	-	-	-	0.06±0^{g}	-	-	0.17±0^{JKLM}	-	0.74±0^{B}	0.38±0^{P}	-
A21	-	-	-	11.92±0^{MN}	-	-	-	0.08±0^{e}	-	-	0.19±0^{H}	0.07±0^{m}	0.72±0^{CD}	0.42±0^{KL}	-
A22	-	-	-	12.23±0.01^{L}	-	-	-	0.09±0^{e}	-	-	0.2±0^{GH}	0.39±0^{X}	0.71±0^{EF}	0.43±0^{K}	-
A23	-	-	-	11.82±0NO	-	-	-	0.19±0^{TUVWXY}	-	-	0.19±0^{H}	0.35±0^{Y}	0.7±0^{FG}	0.45±0^{J}	0.64±0^{KL}
A24	-	-	-	12.49±0.01^{K}	-	-	-	0.08±0^{e}	-	-	0.19±0^{H}	0.68±0^{L}	0.7±0^{G}	0.42±0^{LM}	-
A25	-	-	-	9.14±0.01^{bc}	-	-	-	0.17±0^{Za}	-	-	0.17±0^{JKL}	-	0.69±0^{H}	0.41±0^{MN}	-
A26	-	-	-	6.82±0.01^{m}	-	-	-	0.2±0^{QRS}	0.02±0.03^{V}	-	0.19±0^{H}	0.79±0^{G}	0.52±0^{R}	0.64±0^{B}	0.37±0^{ab}
A27	-	-	-	9.18±0.01^{b}	-	-	0.04±0^{KLMN}	0.19±0^{STUVW}	-	-	0.24±0^{C}	0.68±0^{L}	0.71±0^{DE}	0.4±0°	-
A28	-	-	-	11.76±0.01^{OP}	-	-	-	0.1±0^{d}	-	-	0.21±0^{EF}	0.63±0^{P}	0.72±0^{C}	0.42±0^{LMN}	-
A29	-	-	-	7.7±0.01^{ij}	-	-	-	0.22±0^{KLM}	-	-	0.24±0^{C}	0.78±0^{H}	0.82±0^{A}	0.63±0^{C}	-
A30	-	-	-	9.62±0.01^{Z}	-	-	-	0.23±0^{K}	-	-	0.25±0^{C}	0.8±0.01^{F}	0.51±0^{R}	0.6±0^{D}	-
T1	-	-	1.29±0.27^{OPQRS}	9.69±0^{YZ}	0.01±0^{OPQRSTUV}	0.1±0^{YZab}	0.04±0^{NOP}	0.19±0^{STUV}	0.07±0^{HIJK}	0.06±0^{XYZa}	0.16±0^{LM}	0.72±0^{J}	0.62±0^{L}	0.33±0^{Q}	0.38±0^{Za}
T2	-	-	1.14±0^{STUVW}	1.37±0^{v}	0.01±0^{STUVW}	0.1±0^{Zab}	0.04±0^{LMNO}	0.22±0^{MNO}	0.09±0^{FGH}	0.08±0^{STU}	0.17±0^{KLM}	0.72±0^{J}	0.61±0^{M}	0.31±0^{TU}	0.41±0^{WX}

T3	-	-	1.16±0.01^{STUV}	8.04±0.03^{g}	0.01±0^{NOPQRS}	0.1±0^{XYZa}	0.03±0^{OP}	0.17±0^{Za}	0.06±0^{IJKLM}	0.06±0^{XYZabc}	0.12±0^{QR}	0.58±0^{R}	0.32±0^{b}	0.15±0^{X}	0.34±0^{c}
T4	-	-	1.15±0.01^{STUVW}	1.38±0^{v}	0.01±0^{STUVW}	0.09±0^{Zab}	-	0.21±0^{PQ}	0.06±0^{JKLM}	0.06±0^{XYZabc}	0.09±0^{WXYZ}	0.34±0^{Y}	0.08±0^{kl}	0.11±0^{Y}	0.42±0^{UVW}
T5	-	-	1.01±0^{VWXYZ}	16.01±0^{D}	0.01±0^{UVW}	0.09±0^{abc}	-	0.17±0^{Z}	0.04±0^{OPQRST}	0.05±0^{ef}	0.1±0^{UVWX}	-	0.55±0^{P}	0.29±0^{VW}	0.4±0^{XY}
T6	7.76±0^{G}	-	1.36±0^{MNOPQ}	7.37±0^{k}	0.03±0^{FG}	0.15±0^{OPQR}	0.1±0^{G}	0.35±0^{D}	0.18±0^{C}	0.22±0^{E}	0.18±0^{IJ}	0.72±0^{J}	0.18±0^{f}	0.08±0^{bc}	0.67±0^{I}
T7	8±0^{D}	-	1.52±0^{JKLMN}	11.06±0^{Q}	0.02±0^{GHIJ}	0.14±0^{RST}	0.1±0^{GH}	0.31±0^{F}	0.16±0^{D}	0.2±0^{G}	0.18±0^{IJK}	0.57±0^{S}	0.18±0^{f}	0.07±0^{cd}	0.77±0^{G}
T8	-	-	0.98±0.02^{WXYZ}	7.08±0.01^{l}	0.01±0^{STUVW}	0.12±0^{UVW}	0.03±0^{OP}	0.19±0^{STU}	0.07±0^{HIJK}	0.07±0^{VWX}	0.15±0^{OP}	0.7±0^{K}	0.66±0^{JK}	0.45±0^{J}	0.39±0^{YZ}
T9	-	-	1.01±0^{UVWXYZ}	6.33±0.04^{o}	0.01±0^{OPQRSTU}	0.13±0^{STU}	0.03±0^{P}	0.15±0^{c}	0.05±0^{OPQRS}	0.05±0^{bcde}	0.12±0^{Q}	0.53±0^{U}	0.33±0^{a}	0.29±0^{W}	0.28±0^{f}
T10	-	-	1.17±0.01^{STUV}	9.94±0^{WX}	0.01±0^{OPQRSTU}	0.11±0^{XYZ}	0.03±0^{OP}	0.18±0^{WXY}	0.07±0.01^{IJKL}	0.07±0^{WX}	0.15±0.01^{OP}	0.67±0^{LM}	0.6±0^{M}	0.32±0^{QRS}	0.47±0^{R}
T11	-	-	1.02±0.04^{UVWXYZ}	6.13±0^{p}	0.01±0^{TUVW}	0.11±0^{WXY}	-	0.23±0^{KL}	0.07±0^{GHIJK}	0.06±0^{XYZabc}	0.14±0^{P}	0.67±0^{MN}	0.66±0^{J}	0.48±0^{FG}	0.36±0^{b}
T12	-	-	1.17±0^{RSTUV}	8.04±0.04^{g}	0.02±0^{MNOPQR}	0.14±0^{QRST}	-	0.17±0^{Za}	0.04±0^{OPQRST}	0.06±0^{abcd}	0.12±0^{QRS}	-	0.35±0^{Y}	0.32±0^{QR}	0.48±0^{Q}
T13	-	-	-	5.23±0.03^{s}	-	-	0.15±0^{CD}	-	0.05±0^{MNOP}	0.08±0^{PQRS}	0.02±0^{h}	0.3±0^{Z}	-	-	-
T14	10.96±0^{B}	-	1.67±0.03^{GHIJ}	12.39±0^{K}	0.03±0^{EF}	0.22±0^{GHI}	0.05±0^{K}	0.46±0^{B}	0.27±0^{A}	0.3±0^{A}	0.27±0.01^{B}	0.78±0^{H}	0.33±0^{Za}	0.1±0^{YZ}	1.37±0^{B}
T15	7.23±0^{I}	-	1.44±0^{LMNO}	9.4±0.23^{a}	0.02±0^{GHIJ}	0.21±0^{IJK}	0.08±0^{I}	0.29±0^{G}	0.16±0^{D}	0.19±0^{GH}	0.2±0^{EFG}	0.57±0^{S}	0.15±0^{gh}	0.06±0^{de}	0.77±0^{G}

T16	6.01±0^{K}	-	1.35±0.01^{NOPQR}	8.34±0.02^{e}	0.02±0^{KLMN}	0.16±0^{LMN}	0.08±0^{I}	0.28±0^{H}	0.15±0^{D}	0.19±0^{H}	0.16±0^{LM}	0.47±0^{W}	0.15±0^{h}	0.05±0^{e}	0.77±0^{G}
T17	7.78±0^{F}	-	1.46±0^{KLMNO}	10.4±0.06^{T}	0.04±0^{CD}	0.2±0.01^{K}	0.09±0^{GHI}	0.35±0^{D}	0.19±0^{C}	0.21±0^{EF}	0.21±0^{E}	0.72±0^{J}	0.18±0^{f}	0.07±0^{cd}	0.59±0^{N}
T18	-	-	1.13±0.04^{STUVW}	9.69±0.02^{YZ}	0.01±0^{NOPQRST}	0.1±0^{XYZa}	-	0.21±0^{OP}	0.06±0^{KLMN}	0.06±0^{XYZabc}	0.14±0^{OP}	0.66±0^{N}	0.59±0^{N}	0.3±0^{UV}	0.3±0^{ef}
T19	-	-	0.87±0.02^{Z}	6.44±0.03^{o}	0.01±0^{RSTUVW}	0.07±0^{d}	-	0.15±0^{c}	0.03±0^{STU}	0.03±0^{g}	0.07±0^{bcde}	-	0.04±0^{r}	0.1±0^{YZa}	0.2±0^{h}
T20	-	-	-	5.45±0^{r}	-	-	0.15±0.01^{CD}	-	0.05±0^{LMNOP}	0.08±0^{STU}	0.02±0^{h}	0.26±0^{c}	-	-	-
T21	-	-	-	4.18±0^{u}	-	-	0.11±0^{F}	-	0.03±0^{TUV}	0.05±0^{cdef}	0.01±0^{i}	0.02±0^{n}	-	-	-
T22	7.75±0^{G}	-	1.37±0.02^{MNOP}	11.66±0^{P}	0.02±0^{GHIJ}	0.15±0^{NOPQ}	0.09±0^{GH}	0.26±0^{I}	0.14±0^{E}	0.17±0^{I}	0.2±0^{EFG}	0.51±0^{V}	0.13±0^{i}	0.05±0^{e}	0.67±0^{I}
T23	8.86±0^{C}	-	1.81±0^{FG}	11.97±0^{M}	0.03±0^{EF}	0.18±0^{L}	0.04±0.01^{KLMNO}	0.4±0^{C}	0.22±0^{B}	0.24±0^{D}	0.24±0^{C}	0.82±0^{E}	0.26±0^{d}	0.07±0^{bc}	0.81±0^{E}
T24	-	-	0.92±0^{YZ}	7.47±0.04^{k}	0.01±0^{VW}	0.13±0^{RST}	0.03±0^{OP}	0.16±0^{b}	0.06±0^{LMNO}	0.06±0^{YZabc}	0.12±0^{Q}	0.55±0^{T}	0.52±0^{R}	0.29±0^{W}	0.43±0^{TUV}
T25	-	-	1.07±0.01^{TUVWXY}	7.62±0.01^{j}	0.01±0^{NOPQRST}	0.16±0^{MNOP}	-	0.17±0^{Za}	0.05±0^{OPQRST}	0.06±0^{YZabc}	0.09±0^{XYZa}	-	0.07±0^{opq}	0.11±0^{Y}	0.58±0°
T26	-	-	1.19±0.02^{QRSTU}	9.82±0^{XY}	0.02±0^{LMNOPQ}	0.16±0.01^{MNO}	-	0.16±0^{ab}	0.05±0^{OPQRST}	0.06±0^{abcd}	0.1±0^{VWXY}	-	0.06±0^{pq}	0.11±0^{YZ}	0.69±0^{H}
T27	-	-	1.1±0^{TUVWX}	8.24±0^{ef}	0.01±0^{QRSTUV}	0.15±0.01^{MNOP}	-	0.18±0^{XY}	0.06±0^{LMNOP}	0.06±0^{YZabc}	0.12±0^{QR}	-	0.55±0^{Q}	0.31±0^{STU}	0.61±0^{M}
T28	-	-	1.14±0.02ST	9.04±0.03^{c}	0.01±0^{NOPQRS}	0.15±0^{NOP}	-	-	0.05±0^{OP}	0.06±0^{Za}	0.09±0^{W}	-	0.07±0no	0.11±0^{Y}	0.66±0^{IJ}

			UVW						QRST	bcd	XYZa			Z	
T29	-	-	0.93 ± 0^{XYZ}	6.64 ± 0^{n}	0.01 ± 0^{NOPQRST}	0.11 ± 0.01^{WX}	-	0.22 ± 0^{KLM}	0.07 ± 0^{HIJK}	0.06 ± 0^{XYZab}	0.11 ± 0^{RST}	0.61 ± 0^{Q}	0.38 ± 0^{X}	0.32 ± 0^{QR}	0.42 ± 0^{VW}
T30	-	-	1.14 ± 0^{STUVW}	8.36 ± 0.03^{e}	0.01 ± 0^{NOPQRS}	0.13 ± 0^{TUV}	-	0.18 ± 0^{WXY}	0.06 ± 0^{LMNOP}	0.06 ± 0^{YZabc}	0.11 ± 0^{STU}	-	0.33 ± 0^{Z}	0.3 ± 0^{UV}	0.61 ± 0^{MN}
T31	-	-	-	5.72 ± 0^{q}	-	-	0.12 ± 0^{F}	-	0.04 ± 0^{PQRST}	0.07 ± 0^{UVW}	0.02 ± 0^{h}	0.34 ± 0^{Y}	-	-	-
T32	7.8 ± 0^{E}	-	1.54 ± 0^{JKLM}	8.26 ± 0^{ef}	0.03 ± 0^{GH}	0.22 ± 0^{EFGH}	0.04 ± 0^{KL}	0.4 ± 0^{C}	0.23 ± 0^{B}	0.25 ± 0^{C}	0.2 ± 0^{GH}	0.57 ± 0^{S}	0.21 ± 0^{e}	0.07 ± 0^{bc}	1.44 ± 0^{A}
T33	-	-	1.01 ± 0^{VWXYZ}	7.77 ± 0^{hi}	0.01 ± 0^{VW}	0.11 ± 0^{WX}	0.03 ± 0^{OP}	0.22 ± 0^{KLM}	0.09 ± 0^{FGH}	0.07 ± 0^{TUVW}	0.15 ± 0NO	0.07 ± 0^{m}	0.67 ± 0^{I}	0.45 ± 0^{IJ}	0.48 ± 0^{Q}
T34	-	0.65 ± 0^{D}	-	6.37 ± 0°	-	-	0.16 ± 0^{C}	-	0.05 ± 0^{LMNOP}	0.08 ± 0^{STUV}	0.02 ± 0^{h}	0.31 ± 0^{Z}	-	-	-
T35	-	-	0.87 ± 0.02^{Z}	6.15 ± 0^{p}	-	0.11 ± 0.01^{VWX}	-	0.17 ± 0^{Za}	0.04 ± 0^{OPQRST}	0.05 ± 0^{ef}	0.08 ± 0^{abcd}	-	0.06 ± 0^{pq}	0.1 ± 0^{YZa}	0.43 ± 0ST
T36	-	-	1.13 ± 0^{STUVW}	8.84 ± 0^{d}	0.01 ± 0^{NOPQRST}	0.1 ± 0^{YZab}	0.04 ± 0^{MNOP}	0.16 ± 0^{ab}	0.06 ± 0^{LMNOP}	0.05 ± 0^{ef}	0.14 ± 0^{OP}	0.66 ± 0^{N}	0.67 ± 0^{I}	0.42 ± 0^{KL}	0.38 ± 0^{YZa}
T37	-	-	1.05 ± 0^{TUVWXYZ}	7.41 ± 0^{k}	0.01 ± 0^{W}	0.09 ± 0.01^{bcd}	-	0.17 ± 0^{Z}	0.04 ± 0^{OPQRST}	0.04 ± 0^{f}	0.11 ± 0^{TUV}	0.15 ± 0^{gh}	0.06 ± 0^{q}	0.09 ± 0^{a}	0.37 ± 0^{Za}
T38	-	-	1.09 ± 0^{TUVWXY}	7.89 ± 0^{h}	0.01 ± 0^{STUVW}	0.14 ± 0.01^{PQRS}	-	0.19 ± 0^{VWXY}	0.05 ± 0^{LMNOP}	0.05 ± 0^{def}	0.1 ± 0^{UVWX}	0.34 ± 0^{Y}	0.07 ± 0^{mno}	0.1 ± 0^{Za}	0.32 ± 0^{d}
T39	-	-	1.16 ± 0^{STUV}	1.39 ± 0^{v}	0.01 ± 0^{QRSTUV}	0.09 ± 0.01^{Zabc}	-	0.22 ± 0^{MNO}	0.06 ± 0^{JKLM}	0.06 ± 0^{YZabc}	0.13 ± 0^{Q}	0.65 ± 0°	0.65 ± 0^{K}	0.32 ± 0^{RST}	0.31 ± 0^{de}
T40	-	-	-	6.07 ± 0^{p}	-	-	0.13 ± 0^{E}	-	0.05 ± 0^{LMNOP}	0.09 ± 0°	0.02 ± 0^{h}	0.17 ± 0^{f}	-	-	-
T41	-	-	-	4.65 ± 0^{t}	-	-	0.18 ± 0^{B}	-	0.04 ± 0^{QR}	0.06 ± 0^{W}	0.02 ± 0^{h}	0.16 ± 0^{fg}	-	-	-

									STU	XY					
T42	6.68±0.02^{J}	0.77±0^{A}	-	8.37±0.01^{e}	0.06±0^{A}	0.01±0^{ef}	-	-	-	0.17±0^{I}	-	-	-	-	-
T43	4.46±0^{L}	0.65±0^{C}	-	5.58±0^{r}	0.06±0^{B}	0.02±0^{e}	0.18±0.01^{AB}	-	-	0.12±0^{L}	-	0.26±0^{c}	-	-	-
T44	-	0.69±0^{B}	-	7.86±0^{h}	-	0.02±0^{e}	0.18±0^{A}	-	0.06±0^{IJKLM}	0.11±0^{M}	0.02±0^{h}	0.28±0^{b}	-	-	-
T45	-	-	1.22±0.35^{PQRST}	15.05±0^{E}	0.01±0^{STUVW}	0.08±0.02^{cd}	-	0.24±0^{J}	0.09±0^{F}	0.07±0^{VWX}	0.16±0^{MN}	0.76±0^{I}	0.47±0^{T}	0.48±0^{F}	0.51±0^{P}
T46	11.6±0^{A}	-	1.63±0.03^{HIJK}	10.14±0.01^{UV}	0.03±0^{GH}	0.17±0^{L}	0.05±0^{K}	0.48±0^{A}	0.26±0^{A}	0.26±0^{B}	0.25±0^{C}	1.01±0^{B}	0.27±0^{c}	0.08±0^{b}	1.43±0^{A}
T47	-	-	-	5.58±0^{r}	-	-	0.15±0.01^{D}	-	0.04±0^{OPQRST}	0.09±0^{OPQR}	0.02±0^{h}	0.24±0^{d}	-	-	-
T48	7.41±0^{H}	-	1.57±0.02^{IJKL}	14.83±0.03^{F}	0.03±0^{E}	0.17±0.01^{LM}	0.09±0^{HI}	0.3±0^{G}	0.15±0^{D}	0.21±0^{F}	0.2±0^{FG}	0.61±0^{Q}	0.16±0^{g}	0.06±0^{de}	0.87±0^{D}
T49	-	-	1.02±0^{UVWXYZ}	7.1±0^{l}	0.01±0^{RSTUVW}	0.15±0^{OPQR}	-	0.2±0^{RSTU}	0.06±0^{LMNO}	0.06±0^{abcd}	0.14±0^{OP}	0.56±0^{S}	0.58±0°	0.31±0^{RSTU}	0.24±0^{g}
T50	-	-	1.17±0^{RSTUV}	8.17±0^{fg}	0.01±0^{PQRSTUV}	0.16±0^{MNOP}	-	0.19±0^{STUVWX}	0.06±0^{LMNO}	0.06±0^{WXYZ}	0.09±0^{XYZa}	-	0.08±0^{klm}	0.11±0^{Y}	0.79±0^{F}

[a] The data was represented as "mean ± standard deviation (SD)". Values with no same letters within each column were significantly different ($p < 0.05$).

[b] "-" was represented as "no detected (ND)".

[c] The codes of samples were named as the uppercase of the first letter of AT and TT followed a number, respectively.

AT: ancient tea; TT: terrace tea; WE: water extract; TPC: total phenolics; TFAAs: total free amino acids; EGC: (-)-epigallocatechin; C: (+)-catechin; EGCG: (-)-epigallocatechin gallate; EC: (-)-epicatechin; GCG: (-)-gallocatechin gallate; ECG: (-)-epicatechin gallate; Asp: aspartic acid; Ser: serine; Glu: glutamic acid; Gly: glycine; Ala: alanine; Cys: cysteine; Val: valine; Ile: isoleucine; Leu: leucine; Tyr: tyrosine; Phe: phenylalanine; Lys: lysine; His: histidine; Arg: arginine.

Table S2 The model scores of AT and TT based on Eq.(1) and Eq.(2)

Code[a]	Model scores (1)	Model scores (2)	Code[a]	Model scores (1)	Model scores (2)
A1	2.368922083	-0.434311529	T1	-4.010137206	-4.71089581
A2	2.877113246	-0.569574172	T8	-3.17002888	-3.700796255
A7	2.98396923	-0.041798414	T18	-4.26061643	-5.176785268
A3	2.481027853	0.425092374	T9	-2.76558328	-3.189558442
A4	3.47068088	0.44530769	T36	-3.661759478	-4.312483509
A5	4.651351567	0.022148544	T2	-4.534144088	-4.526782926
A8	3.67147147	-0.252240833	T3	-2.520334128	-4.041577575
A9	3.771261762	1.459796624	T10	-2.472622621	-4.444159457
A10	4.284870922	1.734657245	T19	-2.828913666	-3.876711224
A11	1.848951885	0.162755639	T24	-3.877034891	-5.439068586
A6	7.715111632	4.906151609	T25	-1.608089812	-3.240837705
A12	3.127642528	0.055126101	T26	-2.225420556	-4.159987011
A13	0.720423034	-0.850226583	T49	-1.794187217	-2.694627026
A14	2.572416023	0.661518183	T27	-4.153928339	-4.942697383
A15	3.92520325	-0.004995694	T33	-1.026615522	-1.960151119
A16	1.777477025	-0.588694507	T28	-4.127494963	-4.945896987
A17	3.464580326	0.35719645	T11	-2.322459976	-2.92271353
A18	5.540269958	1.55198362	T37	-3.21329231	-4.11617879
A19	4.364257802	1.976487197	T29	-3.125797387	-3.905847497
A20	3.612509264	1.888638018	T38	-2.212195363	-3.062395785
A21	3.579891724	1.647595337	T39	-4.006567856	-3.505782576
A22	3.475688132	1.490248504	T4	-3.644609454	-3.598450679
A23	6.398827044	4.164551132	T50	-3.225836523	-4.439039053

A24	6.686465147	3.73433455	T30	-2.475419256	-3.813464357
A25	5.334546992	3.110535199	T12	-2.466087604	-3.322584672
A26	5.999756625	3.709542823	T42	-1.593494019	-3.441127799
A27	9.484750659	7.42260526	T43	-2.806748675	-4.043139091
A28	5.341454135	2.605570395	T44	-3.202976616	-3.565561341
A29	5.067905552	3.433488605	T34	-2.682258193	-4.086851451
A30	7.39610774	5.488806807	T47	-2.389411296	-3.562563226
			T20	-2.56218295	-3.681491474
			T21	-3.580187792	-4.357203841
			T13	-2.007612581	-3.268676597
			T31	-1.790343238	-2.498719617
			T40	-2.162662094	-3.592919249
			T41	-2.387921662	-2.847463568
			T45	-0.88416839	-3.250922493
			T5	-1.209689919	-3.847638926
			T35	-2.041355989	-2.525991595
			T48	-2.467367936	-4.633077079
			T6	-2.828046486	-3.400088717
			T7	-2.755359992	-4.090102094
			T14	-1.849820095	-3.514052032
			T22	-2.073932708	-3.4660237
			T15	-2.883837039	-3.83948177
			T16	-2.663267871	-3.437814814
			T17	-2.354055512	-3.548474442
			T46	-2.672342582	-3.59985118
			T32	-3.162364858	-4.029138391

			T23	-1.997534169	-3.0904262
Max	9.484750659	7.42260526	Max	-0.88416839	-1.960151119
Min	0.720423034	-0.850226583	Min	-4.534144088	-5.439068586

[a] The codes of samples were named as the uppercase of the first letter of AT and TT followed a number, respectively.

AT: anceint tea; TT: terrace tea.
